# Older Adults’ Behaviors and Preferences for Seeking Sexual Health Information

**DOI:** 10.1080/19317611.2025.2527050

**Published:** 2025-07-06

**Authors:** Louise Bourchier, Helen Bittleston, Sue Malta, Meredith Temple-Smith, Jane S. Hocking

**Affiliations:** a Melbourne School of Population and Global Health, The University of Melbourne, Melbourne, Australia; b Department of General Practice and Primary Care, The University of Melbourne, Melbourne, Australia

**Keywords:** Older adults, sexual health, health promotion, primary care

## Abstract

**Objectives:**

Sexual expression is important to many older adults, but this population may be overlooked by sexual health campaigns and services. This study sought to understand the sexual health information-seeking behaviors and preferences of older adults, including whether and where they seek this information, the characteristics associated with seeking it, as well as satisfaction, preferences, and reasons for not seeking it.

**Methods:**

The data were gathered in 2021 via a cross-sectional online survey of Australians aged 60 and over. There were seven quantitative outcomes and one set of free-text comments. Quantitative outcomes were analyzed using descriptive statistics, chi^2^ test, and logistic regression. The free-text comments were analyzed using qualitative content analysis.

**Results:**

The survey sample was comprised of 1,470 respondents with an equal balance of men (49.9%) and women (49.7%) and a median age of 69 years (range of 60–92 years). Findings showed that 41.2% (602/1,461; 95%CI 38.7–43.7) had sought information, and 63.6% were satisfied with the information found. Being male, STI testing, online dating, age 70–79, and urban-living were associated with information-seeking. Healthcare providers were the most utilized and trusted information source, and many respondents were willing to look online. One in five did not seek information when they needed it, outlining various barriers preventing them from doing so.

**Conclusions:**

Many older adults seek sexual health information, and with some experiencing access barriers and one-third unsatisfied, there is room for improvement. Relevant, accessible information should be provided by healthcare professionals and credible websites.

## Introduction

Concepts of “healthy aging” focus increasingly on the aspects of life that older adults subjectively value, moving away from models of decline, vulnerability, and risk, to a strengths-based perspective supporting older adults to continue the activities that bring them the most meaning and vitality (Menassa et al., 2023). Sexual expression can be one such important source of physical and emotional well-being, that many (though not all) older adults value as an important part of their lives (James et al., 2023; Roman Lay et al., 2023; Sinković & Towler, 2019; Stahl et al., 2019; Stentagg et al., 2021; Towler et al., 2022). The UN Decade of Healthy Aging 2021–2030 (WHO, 2020), with its emphasis on holistic well-being, provides an opportunity to highlight older adults’ sexual health as an aspect of well-being that needs further attention.

Adopting the WHO’s working definition, “sexual health” encompasses all aspects of sexual rights and well-being (WHO, 2006). People may face a range of sexual challenges as they age, for example maintaining sexual activity while living with chronic illnesses, continuing sexual activity after cancer or surgery, beginning dating after divorce or death of a partner, adapting to age-related changes in physiology, such as vaginal dryness and erection difficulties, and navigating intimacy when one or both partners are in aged care. Older adults (defined in this paper as those aged 60 and over) may benefit from information to help maintain and improve their sexual lives as they manage these changes and challenges. Currently, however, little is known about whether older adults are able to access sexual health information when they need it, their information-seeking behaviors, or the types of resources older users find useful and acceptable. In this paper, we explore these issues with a lens on the Australian context.

While older people may want information to support their sexual health, most sexual health information is targeted to young people and groups at higher risk of sexually transmissible infections (STIs), there is little aimed at older age groups. Although STI prevalence in Australia is lower among older adults than among younger people, diagnosis rates are increasing, and STI knowledge and testing rates are lower among older age groups, indicating a potentially unmet need for sexual health information and health promotion materials targeted to older demographics (Bourchier et al., 2020; Heywood et al., 2017; Kirby Institute, 2024; Lyons et al., 2017).

Research from Australia and elsewhere has established that some older adults do seek sexual health information (Fileborn et al., 2017; Lyons et al., 2018). Questions on sexual health information-seeking behaviors were included in “Sex, Age, and Me,” a large mixed methods study of the sexual health and behaviors of Australians aged 60 and over conducted in 2015. They found that, among heterosexual participants potentially at risk of an STI, 12.4% of men and 14.9% of women had sought STI information in the previous year, and that media (magazines, TV) was the main source of STI information, followed by GPs/healthcare providers and the internet (Lyons et al., 2018). In terms of the resources they trusted, participants relied most on GPs/healthcare providers followed by media sources (Lyons et al., 2018). In the qualitative arm of the study, 53 participants were interviewed about their experiences accessing sexual health information (Fileborn et al., 2017). The internet was a key source of information for these individuals, who described being discerning in gauging the trustworthiness of internet sources, preferring government, medical, or university sources (Fileborn et al., 2017). While Sex, Age and Me established that older Australians do indeed seek sexual health information, the main places they seek it, and the sources they trust most, there are still gaps in our understanding. The characteristics of older adults who are more or less likely to seek this information, whether they find adequate answers to their sexual health questions, and why others do not seek sexual health information are issues yet to be explored and the answers to these questions may be useful in planning health promotion efforts targeted to these populations.

To design effective sexual health initiatives that meet the needs of older users, it is necessary to understand whether and how they want to access this information, who is most and least likely to seek it and where from, barriers that may prevent some older users from seeking sexual health information, as well as understanding whether the information currently available is suitable. By asking older adults directly, we can gain an idea of what “best practice” looks like for sexual healthcare and health promotion among older populations, acknowledging that this may differ from services and campaigns targeting younger people. In 2021, we ran the “SHAPE2” (Sexual Health Aging Perspectives and Education) survey to shed light on these questions. In this paper, we explore the sexual health information-seeking behaviors and preferences of Australians aged 60 and over and investigate socio-demographic and behavioral factors associated with information-seeking. These findings will inform future sexual health initiatives targeting older adults to ensure resources and formats meet the specific needs of older users.

## Materials and methods

### SHAPE2 survey and recruitment

For comprehensive information on the study design, recruitment, and full schedule of survey questions, as well as details of the sample obtained, please see our published Methods paper (Bourchier et al., 2023). In brief, we conducted the SHAPE2 cross-sectional online survey between April and September 2021. Eligible participants were aged 60 years old and over and living in Australia. The survey took ∼17 min to complete and contained 63 questions. Participants were asked about sexual health information they had sought since turning 60, and their preferences and future intentions around sexual health information-seeking, as well as demographic questions. Participants were recruited via emails sent to relevant organizations and community groups, and via paid Facebook advertising. No incentives were offered for participation.

### Ethical approval

Ethics approval was obtained from the University of Melbourne Human Research Ethics Committee (HREC ID 2057393). Apart from eligibility and consent questions, all questions were optional, and participants were able to exit the survey at any time. Participants were anonymous and not asked identifying questions, with duplicates identified via IP addresses and removed during data cleaning (see Bourchier et al., 2023 for more detail).

### Data management and analysis

We had seven quantitative variables of interest as follows:

1. *Whether or not they had sought sexual health information for themselves since turning 60—binary variable (Yes/No).*
2. *Source(s) of sexual health information, the last time they looked—a multi-choice variable where multiple sources could be selected.*
3. *Whether those who had sought sexual health information had their questions answered sufficiently—categorical variable (Yes/No/Partially, but not completely/I don’t know)*
4. *Whether participants would use the internet to look for sexual health information in future—binary variable (Yes/No)*
5. *Which internet sources they would consider using to search for sexual health information in future (asked only of those who indicated they would use the internet in the future)—categorical variable (measured using a 5-point Likert scale of Extremely Likely to Extremely Unlikely).*
6. *Which non-internet sources they would consider using to search for sexual health information in future—categorical variable (measured using a 5-point Likert scale of Extremely Likely to Extremely Unlikely).*
7. *What criteria participants would use to gauge trustworthiness of sexual health information sources—categorical variable (measured using a 5-point Likert scale of Extremely Important to Extremely Unimportant).*

We used univariable and multivariable logistic regression models to explore factors associated with having sought sexual health information since turning 60 (variable 1). Exposure variables were categorized as follows:

- Gender—Female/Male (gender diverse participants excluded due to small number (*n* < 10)
- Age—60–69 years/70–79 years/80+ years
- Location—Major cities/Regional and remote areas (using Australian Bureau of Statistics (ABS) postcode classifications)
- Sexual orientation—Heterosexual/Homosexual and bisexual (including queer and pansexual)/Something else (including those who said asexual or described their sexuality in another way)
- Relationship and sexual activity—In a relationship and sexually active/In a relationship and not sexually active/Single and sexually active/Single and not sexually active
- Online dating experience—Not used since turning 60/Used since turning 60
- History of testing and infection with an STI—No STI test since turning 60/Negative STI test(s) since turning 60/Diagnosed with an STI since turning 60

The remaining outcomes were explored using descriptive statistics. Given the gender difference in sexual health information-seeking identified in the first analysis (variable 1), when analyzing sources of information (variable 2), we compared proportions between males and females using Chi^2^ test. For all variables using Likert scales, the top two categories were combined to describe the items most “likely” or “important,” variables were then ranked to identify the top three variables. All quantitative analyses were performed using Stata 16.0 (StataCorp, College Station, TX, USA). Denominators vary between variables as most survey questions were optional, and some questions were only displayed to a sub-set of participants using skip-logic based on previous responses.

We also analyzed one free-text outcome (outcome 8) which investigated participants’ reasons for not seeking sexual health information (asked only of those who had not sought sexual health information since turning 60) using qualitative content analysis (Forman & Damschroder, 2007; Hsieh & Shannon, 2005). Comments were organized into themes and sub-themes, grouping together the reasons given by the participants. Analysis was conducted by LB and verified by SM.

## Results

### Sample

A total of 1,470 participants were recruited. Participant characteristics are outlined in Table 1. Ages ranged from 60 to 92 years with a median age of 69 years. Gender was evenly spread between female (49.7%) and male (49.9%) participants. Participants were recruited from all States/Territories of Australia, with two-thirds living in major cities (63.8%) and the remaining third in regional and remote areas. The majority were heterosexual (85.2%) and retired (65.6%). 53.6% were in a relationship and sexually active, 24.1% were in a relationship and not sexually active, and 22.4% were single. One quarter had used online dating before (25.5%), and 8.1% had had an STI test since turning 60.

**Table 1.** Characteristics of participants.

Characteristics		Number of participants*	%
Gender (*N* = 1,470)	Male	734	49.9
	Female	730	49.7
	Other gender identities	6	0.4
Age (years) (*N* = 1,470)	60–64	455	31.0
	65–69	387	26.3
	70–74	321	21.8
	75–79	187	12.7
	80–84	93	6.3
	85+	27	1.8
State/territory (*N* = 1,470)	New South Wales	393	26.7
	Victoria	372	25.3
	Queensland	197	13.4
	Western Australia	174	11.8
	Australian Capital Territory	154	10.5
	South Australia	110	7.5
	Tasmania	58	4.0
	Northern Territory	12	0.8
Location (*N* = 1,456)	Metro	929	63.8
	Regional	510	35.0
	Remote	17	1.2
Employment status (*N* = 1,467)	Retired	963	65.6
	Working (full-time, part-time, or casual)	398	27.1
	Other	106	7.2
Sexual orientation (*N* = 1,085)	Heterosexual	924	85.2
	Homosexual	76	7.0
	Bisexual/pansexual	52	4.8
	Asexual	12	1.1
	Something else	21	1.9
Relationship and sexual activity (*N* = 1,013)	In a relationship and sexually active	543	53.6
	In a relationship and not sexually active	244	24.1
	Single and sexually active	35	3.5
	Single and not sexually active	191	18.9
Online dating (*N* = 1,060)	Never used online dating	790	74.5
	Used online dating, but not since turning 60	129	12.2
	Used online dating since turning 60	141	13.3
Sexually transmissible infections (STIs) (*N* = 1,045)	No STI test since turning 60	960	91.9
	Negative STI test since turning 60	61	5.8
	Diagnosed with an STI since turning 60	24	2.3

* Numbers will not always add up to 1,470 due to some missing values.

### Looking for sexual health information since turning 60

Approximately two in five participants (602/1461, 41.2%; 95%CI: 38.7–43.7) had looked for sexual health information since turning 60 (Table 2). Participants had a greater odds of seeking sexual health information if they were male (adjusted odds ratio [aOR] = 2.0; 95%CI: 1.5–2.6), were aged 70–79 years compared with 60–69 years (aOR = 1.5; 95%CI: 1.1–2.0), had used online dating since turning 60 (aOR = 1.8; 95%CI: 1.1–2.8), or had had a STI test since turning 60 with either a negative (aOR = 2.2; 95%CI: 1.2–4.3) or positive (aOR = 9.5; 95%CI: 2.1–43.2) test result. The odds of having sought sexual health information were reduced if they lived in a regional or remote area as compared to an urban area (aOR = 0.7; 95%CI: 0.6–1.0), or if they were single and not sexually active *versus* those who were in a relationship and sexually active (aOR = 0.6; 95%CI: 0.4–0.9).

**Table 2.** Participants who sought sexual health information for themselves since turning 60.

	Sought sexual health information for self since turning 60		Univariable logistic regression			Multivariable logistic regression*		
	*n*/N	%	OR	95%CI	*p*	aOR	95%CI	*p*
Total sample (*N* = 1,461)	602/1461	41.2	–	–	–	–	–	–
Gender (*N* = 1,456)								
Female	222/726	30.6	Ref	–	–	Ref	–	–
Male	376/730	51.5	2.4	2.0–3.0	<0.001	2.0	1.5–2.6	<0.001
Age (*N* = 1,461)								
60–69	318/839	37.9	Ref	–	–	Ref	–	–
70–79	244/505	48.3	1.5	1.2–1.9	<0.001	1.5	1.1–2.0	0.008
80+	40/117	34.2	0.9	0.6–1.3	0.437	0.9	0.5–1.6	0.764
Location (*N* = 1,447)								
Major cities	399/922	43.3	Ref	–	–	Ref	–	–
Regional and remote	196/525	37.3	0.8	0.6–1.0	0.027	0.7	0.6–1.0	0.039
Sexual orientation (*N* = 1,081)								
Heterosexual	409/920	44.5	Ref	–	–	Ref	–	–
Homosexual/bisexual	63/131	48.1	1.2	0.8–1.7	0.434	0.9	0.6–1.5	0.777
Something else	11/30	36.7	0.7	0.3–1.5	0.400	0.9	0.4–2.1	0.774
Relationship and sexual activity (*N* = 1,009)								
In a relationship and sexually active	251/540	46.5	Ref	–	–	Ref	–	–
In a relationship and not sexually active	110/244	45.1	1.0	0.7–1.3	0.716	0.8	0.6–1.2	0.311
Single and sexually active	20/35	57.1	1.5	0.8–3.1	0.224	0.8	0.4–1.7	0.523
Single and not sexually active	64/190	33.7	0.6	0.4–0.8	0.002	0.6	0.4–0.9	0.008
Online dating (*N* = 1,056)								
Not used online dating since turning 60	392/915	42.8	Ref	–	–	Ref	–	–
Used online dating since turning 60	83/141	58.9	1.9	1.3–2.7	<0.001	1.8	1.1–2.8	0.015
Sexually transmissible infections (*N* = 1,041)								
No STI test since turning 60	411/956	43.0	Ref	–	–	Ref	–	–
Negative STI test since turning 60	39/61	63.9	2.4	1.4–4.0	0.002	2.2	1.2–4.3	0.015
Diagnosed with a STI since turning 60	20/24	83.3	6.6	2.3–19.6	0.001	9.5	2.1–43.2	0.004

Ref: reference category.

* Multivariable logistic regression only includes observations with complete data. *N* = 958.

### Sources of sexual health information

**Figure 1.**
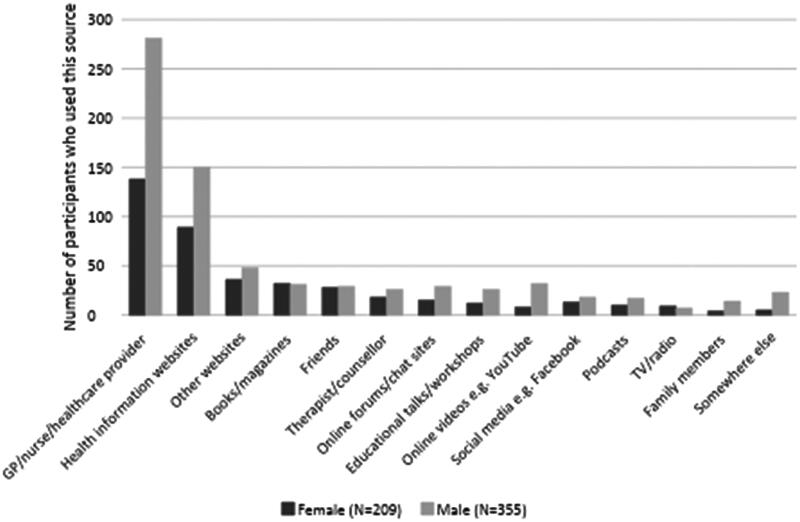
The last place(s) participants sought sexual health information for themselves since turning 60 (*N* = 564)*. *Four gender diverse participants are included in the overall data, but not included in the gender disaggregated data due to small numbers.

Of the participants who had accessed sexual health information since turning 60, most reported the source(s) of information they had last used (568/602, 94.4%). They had used a wide range of sources, with most consulting a GP/nurse/healthcare provider (421/568, 74.1%; 95%CI: 70.5–77.7) and many looking on health information websites (242/568, 42.6%; 95%CI: 38.5–46.7) (Figure 1). Compared with women, men were more likely to have seen a GP/nurse/healthcare provider (79.2 *vs.* 66.0%, *p* = 0.001), or to have watched online videos (9.0 *vs.* 3.8%, *p* = 0.020) and were less likely to have read books/magazines (8.7 *vs.* 15.3%, *p* = 0.017) or to have spoken to a friend (8.2 *vs.* 13.4%, *p* = 0.047).

Most of those who had sought sexual health information indicated whether they found a satisfactory answer to their question (555/602, 92.2%). Nearly two-thirds (353/555, 63.6%; 95%CI: 59.4–67.6) were satisfied that their question had been adequately answered. But over a third were not satisfied with the information they found, they either only partially had their question answered (126/555, 22.7%; 95%CI: 19.3–26.4), did not find satisfactory information (61/555, 11.0%; 95%CI: 8.5–13.9), or were unsure whether they had their question answered (15/555, 2.7%; 95%CI: 1.5–4.4).

### Willingness to use sexual health information sources

Of those who responded to the question asking whether they would be willing to use the internet to seek sexual health information in future, three quarters said they would (1055/1395, 75.6%; 95%CI: 73.3–77.9), with no difference between men (517/694, 74.5%; 95%CI: 71.1–77.7) and women (532/695, 76.5%; 95%CI: 73.2–79.7; *p* = 0.386). The top three online sources participants were most likely to use to look for sexual health information in the future were: (1) Google/search engine, (2) Health information website, and (3) Online videos/YouTube. In terms of non-internet sources, the top three off-line sources participants were most likely to use in future were: (1) GP/nurse/healthcare provider, (2) therapist/counselor, and (3) books/magazines. When gauging trustworthiness of sexual health information sources, the top three criteria most important to participants were: (1) endorsed by a relevant institution (e.g. hospital, university), (2) endorsed by a relevant health organization (e.g. Better Health), and (3) author names and qualifications given. See Appendix A for further details.

### Reason for not seeking sexual health information

Of those who had not looked for sexual health information since turning 60 (*n* = 859), most gave a free-text comment indicating the reason why they had not done so. Of these, 7 comments were excluded for not addressing the question, leaving 695 comments, ranging from one word to several sentences in length. The data were categorized into two themes: (1) Sexual health information not needed; and (2) Impeded from seeking sexual health information, with each theme comprised of several sub-themes. A small number of illustrative quotes are included below, see Appendix B for further details and quotes.

1. ***Sexual health information not needed:*** The majority of participants (558/695, 80.3%) had not sought information because they had not needed it. The reasons given were grouped into four sub-themes. They had not sought information because (i) they were satisfied with their sexual life, (ii) they had not experienced sexual problems, (iii) they felt they were already well informed, or because (iv) they were not sexually active and/or not interested in sex with no indication that they had an unmet need for sexual health information.
  ‘Because our sex life is great’ (Woman, aged 60–64, regional NSW)
  ‘Haven’t had any problems, so didn’t think needed help’ (Man, aged 85+, remote QLD)
  ‘Already well informed’ (Woman, aged 60–64, metro SA)
2. ***Impeded from seeking sexual health information:*** *Approximately 1 in 5 participants (137/695, 19.7%) had not sought sexual health information due to a range of barriers. For these participants, an unmet need for sexual health information was indicated. The reasons given were grouped into seven sub-themes. They had not sought information because (i) they were embarrassed, (ii) they feared the disapproval of others, (iii) they were unsure where to look for information, (iv) they were skeptical about finding answers to their questions, (v) they had given up hope of solving their problems, (vi) sexual concerns had not reached the top of their priorities, and (vii) for other miscellaneous reasons.*
  ‘Probably because of embarrassment…’ (Man, aged 75–79, metro ACT)
  ‘Might be ridiculed and told [I’m] too old to worry about sexual health…’ (Woman, aged 60–64, metro VIC)
  ‘I didn’t know there was helpful information available and I didn’t know the right questions to ask.’ (Woman, aged 60–64, regional VIC)

## Discussion

In this comprehensive study of sexual health information-seeking behaviors and preferences of a substantial survey sample of Australians aged 60 and over, we found that many (although not most) older Australians do look for sexual health information. Results showed that men were more likely than women to have sought this information, being tested for STIs and engaging in online dating were associated with seeking information, and a healthcare provider was the most utilized and most trusted source. This was the first study to investigate why some older adults do not seek sexual health information, identifying that while most do not need it, some experience barriers that prevent them from doing so. With over a third of participants not finding satisfactory answers to their questions, and with one in five hindered from seeking information, our study demonstrates that there is room for improvement in the sexual health support and resources available for older adults.

Our finding that older men were twice as likely as women to have sought sexual health information since turning 60 is similar to that of Sex, Age and Me conducted in Australia six years earlier (Lyons et al., 2018). Men in our study were also more likely than women to have seen a healthcare provider for sexual health information. Our study suggests that either men need more sexual health information than women as they age, or that they are more willing and able to seek out this information than women. The reasons for this discrepancy in information-seeking are unknown with further research needed to explore the causes. One consideration is that older women are less likely to be sexually active than older men (Cismaru-Inescu et al., 2022; Khan et al., 2023; Stentagg et al., 2021), with the lower male-female sex ratio in older age groups a contributing factor (ABS, 2022; United Nations, 2024). This discrepancy highlights different needs and behaviors of older men and women and suggests that sexual health initiatives may need to be targeted by gender. While information-seeking behaviors among gender diverse participants were not analyzed in this study due to a small number of gender diverse participants, the needs of trans and other gender diverse older adults are likely to be distinct, and further research should determine best practice for sexual health initiatives among these populations. Of note was that there were no differences observed between participants of any sexual orientation in terms of sexual health information-seeking behaviors.

Unsurprisingly, people who had been tested for an STI since turning 60 were significantly more likely to seek sexual health information, particularly if they had tested positive. Those who used online dating since turning 60 were also more likely to have sought information. Both findings suggest that older adults who are seeking and/or having sex with new partners have a greater need for sexual health information than those who are not seeking and/or having sex with new partners. This has implications for both health promotion (e.g. targeted information for older users of dating apps) and for healthcare provision (e.g. taking a sexual history so that those with new partners can be offered information and STI testing).

Rural and remote residents were less likely to have sought sexual health information, suggesting either lower availability of information or less motivation to seek it than those in urban areas. People living in rural and remote areas may be less likely to seek information from healthcare providers due to disparities in access to healthcare services and because of perceived lack of confidentiality when living in a small community where there is less opportunity for anonymity—an important consideration with sensitive topics like sexual health (Malatzky & Hulme, 2022; Nic Giolla Easpaig et al., 2022). Disparities in internet access for rural people may also be a factor (Thomas et al., 2023).

Healthcare providers stood out as the most utilized and most trusted source of sexual health information in our study. These findings are similar to Sex, Age and Me, which found healthcare providers the second most common source for STI information after media, and the most relied upon (Lyons et al., 2018). Healthcare providers, especially in primary care, have an important role in addressing older adults’ sexual health needs, even though time constraints, embarrassment, and more urgent health concerns can preclude these conversations (Bourchier et al., 2024; Fileborn et al., 2017; Hinchliff et al., 2021; Malta et al., 2020). Maintaining sexual health and sexual activity—for those who want to—has physical and mental health benefits, so it is important that this topic be addressed by healthcare providers as part of overall care (Fileborn et al., 2015; Liu et al., 2016; Shen & Liu, 2023; Sinković & Towler, 2019; Zhang & Liu, 2020). Initiatives to improve access to sexual health information for older people may include resources that can be given to older patients in medical consultations and also trainings for primary care providers. As STI rates are higher among young people (King et al., 2022), current trainings for clinicians are likely to focus on younger patients, and developing age-inclusive trainings that contextualize sexual healthcare within holistic care through the lifespan may be advisable.

The internet was a key sexual health information source for our participants. Creating and promoting online resources for older adults is likely to be effective for facilitating sexual health information access. However, the findings of both our SHAPE2 survey and Sex, Age and Me emphasize that, to be trustworthy in the eyes of older users, online resources should be created or endorsed by relevant organizations, such as hospitals, universities or government agencies (see also Fileborn et al., 2017; Lyons et al., 2018). Endorsed by The University of Melbourne and the Victorian Government, the “In My Prime” website (https://inmyprime.org.au/) dedicated to older women’s health and well-being provides an example of the type of resources that may be suitable. An important consideration for designing online information is ensuring that websites are easy to find using search terms familiar to older adults, thus, the role of search engine optimization will also be crucial.

Although over half of the participants had not sought sexual health information since turning 60, it is encouraging that most said that the reason for this was that they had not required information, indicating that a substantial amount had no unmet need. However, it is concerning that one in five were hindered from seeking sexual health information. The reasons why offer directions for future health promotion initiatives. To address embarrassment and fear of disapproval, resources should be destigmatizing and age-inclusive emphasizing that sexual health is important to people of all ages (Barrett & Hinchliff, 2018). To reassure older patients that sexual health is a legitimate topic for discussion in the healthcare context, posters could be displayed in clinic waiting rooms, healthcare providers could broach the topic with patients (Hinchliff et al., 2023), and a pre-consultation checklist could prompt discussions (Malta et al., 2018).

The results of this study point to several key recommendations to improve older adults’ access to sexual health information, contributing to the broader goal of improving the health and well-being of older people as envisioned by the UN Decade of Healthy Aging (WHO, 2020).

1. **Encourage healthcare providers to initiate sexual health conversations:** Healthcare providers are encouraged to initiate sexual health conversations with older patients to identify sexual health needs, and to have information on-hand to provide patients. Healthcare providers are advised to broach the topic of sexual health intermittently with their older patients within routine care. Opportunities to discuss sexual health may arise when discussing chronic conditions, medications, and medical events as these can have sexual impacts. See Hinchliff et al. (2023) for further guidance on ways to sexual health conversations.
2. **Develop appropriate online sexual health resources:** Online sexual health resources should be developed that are easy to find, relevant, and appealing to older users. To ensure online resources are trusted by older users, they should be endorsed by hospitals, universities, or government bodies.
3. **Consider gender specific sexual health information:** Effective sexual health promotion for older adults may require resources to be targeted by gender, as men and women have different information-seeking behaviors and needs. Routine screening, such as prostate or breast screening, may provide opportunities for clinicians to broach sexual health. Gender specific health promotion organizations, such as women’s or men’s health organizations, may be well-positioned to address gender specific sexual health needs.
4. **Target older adults with new sexual partners:** Specific resources should be designed for those who are seeking and/or having sex with new partners who may be at elevated with of STIs and may benefit from sexual health information to navigate new sexual relationships. This information should address safer sex and STI testing in particular. Resources could be promoted on dating apps, as the older adults using these apps are actively engaged in seeking out new partners, and this information may therefore be highly relevant to them.
5. **Customize initiatives for rural older adults:** Targeted initiatives are needed for rural older adults to address inequities in sexual health information access. Resources should address barriers and needs unique to the rural context. Health promotion organizations that serve rural communities will be well-positioned to deliver this information due to their understanding of the rural context. Further research may be required to understand how best to deliver education on sensitive topics, such as sexual health in ways that are acceptable to older rural residents.

### Strengths and limitations

A strength of this study is the relatively large number of participants and the inclusion of both quantitative and qualitative data. The sample was fairly representative of the Australian over 60 population in terms of age, gender, and location, within the constraints of a convenience sample. This study builds on earlier research, substantiating their findings, and goes further to explore more nuanced aspects of sexual health information-seeking behaviors and preferences among this population. However, there are some limitations to be considered. Firstly, it was based on a nonrandom sample and, as all questions were optional, there was some missing data, which needs to be considered when interpreting the strength of these results. However, as the main purpose of this research was to explore relationships (e.g. identify characteristics of those more/less likely to seek sexual health information, compare popularity of different information sources) issues of nonrandom sampling and missing data do not undermine the internal validity of the results (Mealing et al., 2010). Secondly, the survey was in English only and administered online, so people from non-English speaking backgrounds and older people (85+ years) who are less likely to use the internet (ABS, 2020) will be under-represented. The results on internet-use for information-seeking may also be inflated as the online survey modality meant that participants already had a certain comfort with using the internet.

## Conclusion

Many older adults value sexual health as part of overall well-being and seek sexual health information to meet their needs but find gaps in accessibility and relevance of the information currently available. Findings from this study offer direction for future sexual health initiatives for older adults, particularly those in Australia. Bolstering sexual conversations and information access through primary care is key, as is providing informative websites that are trusted and easy for older users to find and navigate. Ensuring equity requires seeing older adults not as a homogenous group but by targeting sexual health promotion approaches by gender, location, and sexual behavior. Provision of relevant sexual health information demonstrates respect and acknowledgement of older adults, which will have benefits for overcoming ageism.

## Data Availability

The data that support this study cannot be publicly shared due to ethical or privacy reasons and may be shared upon reasonable request to the corresponding author if appropriate.

## Appendix A

**Table A1.** Willingness to use non-internet sources to seek sexual health information in future.

	Likely to use this source	% (*N* = 1,329)
Talking to a GP/nurse/healthcare provider	936	70.4
Therapist/counselor	549	41.3
Books/magazines	464	34.9
Educational talks/workshops	423	31.8
Friends	344	25.9
TV/radio	233	17.5
Library/community center	213	16.0
Family members	174	13.1

**Table A2.** Willingness to use internet sources to seek sexual health information in future.

	Likely to use this source	% (*N* = 1,018)*
Search for information on Google/search engine	767	75.3
Health information website, e.g. Healthline, Better Health	751	73.8
Online videos, e.g. YouTube	234	23.0
News/current affairs websites	230	22.6
Podcasts	221	21.7
Seniors forum/chat site	191	18.8
Other online forums/chat sites	117	11.5
Pornography websites	103	10.1
Facebook	90	8.8
Blogs, e.g. Sixty and Me	80	7.9
Online dating sites, e.g. RSVP, Lumen	32	3.1
Other social media, e.g. Instagram, Tumblr	26	2.6
Twitter	24	2.4

* Asked only of those who said they were willing to use the internet to look for sexual health information.

**Table A3.** Criteria for determining trustworthiness of sexual health information sources.

	Important for trustworthiness of source	% (*N* = 1,287)
Endorsed by an institution, e.g. hospital, university	935	72.6
Endorsed by a relevant health organization, e.g. Better Health	790	61.4
Author names and qualifications are given	562	43.7
Recent publication or update date	497	38.6
Endorsed by a relevant community org, e.g. seniors advocacy group	400	31.1
Created by people who are 60 and over themselves	333	25.9
Rated and reviewed by users	250	19.4
Visually appealing/looks professional	212	16.5
Need to pay to access it (i.e. is paid more trustworthy free?)	56	4.4

## Appendix B

**Table B1.** Reasons participants had not sought sexual health information: themes and sub-themes with illustrative quotes.

Theme	Sub-theme	Participant quotes
1. Sexual health information not needed (558/695, 80.3%)	(i) Satisfied with sexual life	“Because our sex life is great” (Woman, aged 60–64, regional NSW) “Had new marriage and was happy” (Woman, aged 80–84, metro VIC) “Happily married for over 50 years” (Man, aged 85+, metro QLD)
	(ii) No problems	“All has been good and in working order” (Man, aged 60–64, metro WA) “Because I am still very fit and healthy” (Woman, aged 60–64, metro NSW) “Haven’t had any problems, so didn’t think needed help” (Man, aged 85+, remote QLD)
	(iii) Well informed	“Already well informed” (Woman, aged 60–64, metro SA) “Being a nurse helps with understanding of problems” (Woman, aged 70–74, regional NSW) “I felt I knew all I needed to know” (Man, aged 85+, metro QLD)
	(iv) Not sexually active and/or interested with no unmet need indicated	“Not involved or interested” (Man, aged 80–84, metro ACT) “Post menopause I have no interest and that makes life far less complicated!” (Woman, aged 60–64, regional VIC) “Well, I have not needed information as I don’t have sex” (Man, aged 60–64, metro NSW)
2. Impeded from seeking sexual health information (137/695, 19.7%)	(i) Embarrassment	“Not comfortable speaking with GPs in my region” (Woman, aged 60–64, regional VIC) “Not something I want to share” (Man, aged 60–64, regional TAS) “Probably because of embarrassment…” (Man, aged 75–79, metro ACT)
	(ii) Fear of disapproval	“Might be ridiculed and told [I’m] too old to worry about sexual health…” (Woman, aged 60–64, metro VIC) “…my ‘special friend’ may think there is something wrong with me.” (Woman, aged 60–64, metro QLD) “Probably worried about what GP or others might think (locally)…” (Man, aged 65–69, regional NSW)
	(iii) Unsure where to look	“Did not really know who would be the right person to see for sexual health & well-being after 60! It’s not really publicized very much, is it?” (Woman, aged 60–64, metro VIC) “Don’t know where to go” (Man, aged 70–74, metro ACT) “I didn’t know there was helpful information available and I didn’t know the right questions to ask.” (Woman, aged 60–64, regional VIC)
	(iv) Skeptical about finding answers	“…the belief that I would see it as a waste of time” (Man, aged 75–79, metro ACT) “I don’t expect it to be useful…” (Woman, aged 70–74, metro NSW) “Mostly angled at young people” (Woman, aged 60–64, metro SA)
	(v) Given up hope	“…I talked to my doctor about the complete absence of sexual desire I was experiencing as a result of the lack of estrogen. She prescribed something […] It didn’t work for me. I didn’t want to use HRT […] so I kind of lost heart. I’m totally not interested in any intimacy although I endure it for the sake of my very long term partner.” (Woman, aged 60–64, metro NSW) “Having sex became too fraught with my partner having erectile dysfunction…” (Woman, aged 70–74, metro NSW) “I have not been partnered in over 20 years and have lost hope in ever having another sexual partner.” (Woman, aged 65–69, metro ACT)
	(vi) Not a priority	“Just haven’t got around to it.” (Woman, aged 60–64, metro QLD) “Lazy” (Man, aged 60–64, regional VIC) “Not urgent” (Man, aged 80–84, metro WA)
	(vii) Other reasons	“Cultural expectations that females generally over a certain age are carers, and their needs are irrelevant. The focus is on men’s sexual needs. I’ve never been asked if I have sexual needs. It’s assumed that I don’t!” (Woman, aged 70–74, metro VIC) “Figuring it out by myself” (Man, aged 60–64, metro NSW) “Just thought it was part of growing old” (Man, aged 70–74, regional NSW) “Not certain as I fairly obviously have a problem and am still interested in sex” (Man, aged 85+, metro ACT) “Scared to find out” (Man, aged 65–69, metro NSW)

## References

[CIT0001] Australian Bureau of Statistics (2020). 2018 survey of disability, ageing and carers: Use of information technology. https://www.abs.gov.au/statistics/health/disability/disability-ageing-and-carers-australia-summary-findings/latest-release

[CIT0002] Australian Bureau of Statistics (2022). 3.4 Population counts and age–sex distributions. https://www.abs.gov.au/census/about-census/census-statistical-independent-assurance-panel-report/34-population-counts-and-age-sex-distributions

[CIT0003] Barrett, C., & Hinchliff, S. (2018). *Addressing the sexual rights of older people*. Routledge.

[CIT0004] Bourchier, L., Malta, S., Temple-Smith, M., & Hocking, J. (2020). Do we need to worry about sexually transmissible infections (STIs) in older women in Australia? An investigation of STI trends between 2000 and 2018. *Sexual Health*, *17*(6), 517–524. 10.1071/SH20130 33334416

[CIT0005] Bourchier, L., Temple-Smith, M., Hocking, J. S., & Malta, S. (2024). Older patients want to talk about sexual health in Australian primary care. *Australian Journal of Primary Health*, *30*(4). 10.1071/PY24016 39299683

[CIT0006] Bourchier, L., Temple-Smith, M., Hocking, J., Bittleston, H., & Malta, S. (2023). Engaging older Australians in sexual health research: SHAPE2 survey recruitment and sample. *Sexual Health*, *21*(1), 1–11. 10.1071/SH23116 38071758

[CIT0007] Cismaru-Inescu, A., Hahaut, B., Adam, S., Nobels, A., Beaulieu, M., Vandeviver, C., Keygnaert, I., & Nisen, L. (2022). Sexual activity and physical tenderness in older adults: Prevalence and associated characteristics from a Belgian study. *The Journal of Sexual Medicine*, *19*(4), 569–580. 10.1016/j.jsxm.2022.01.516 35236640

[CIT0008] Fileborn, B., Lyons, A., Heywood, W., Hinchliff, S., Malta, S., Dow, B., Brown, G., Barrett, C., & Minichiello, V. (2017). Talking to healthcare providers about sex in later life: Findings from a qualitative study with older Australian men and women. *Australasian Journal on Ageing*, *36*(4), E50–E56. 10.1111/ajag.12450 28639430

[CIT0009] Fileborn, B., Lyons, A., Hinchliff, S., Brown, G., Heywood, W., & Minichiello, V. (2017). Learning about sex in later life: Sources of education and older Australian adults. *Sex Education*, *17*(2), 165–179. 10.1080/14681811.2016.1273829

[CIT0010] Fileborn, B., Thorpe, R., Hawkes, G., Minichiello, V., Pitts, M., & Dune, T. (2015). Sex, desire and pleasure: Considering the experiences of older Australian women. *Sexual and Relationship Therapy*, *30*(1), 117–130. 10.1080/14681994.2014.936722 PMC4270421 25544829

[CIT0011] Forman, J., & Damschroder, L. (2007). Qualitative content analysis. *Advances in Bioethics*, *11*, 39–62. 10.1016/S1479-3709(07)11003-7

[CIT0012] Heywood, W., Lyons, A., Fileborn, B., Minichiello, V., Barrett, C., Brown, G., Hinchliff, S., Malta, S., & Crameri, P. (2017). Self-reported testing and treatment histories among older Australian men and women who may be at risk of a sexually transmissible infection. *Sexual Health*, *14*(2), 139–146. 10.1071/SH16075 27914483

[CIT0013] Hinchliff, S., Lewis, R., Wellings, K., Datta, J., & Mitchell, K. (2021). Pathways to help-seeking for sexual difficulties in older adults: Qualitative findings from the Third National Survey of Sexual Attitudes and Lifestyles (Natsal-3). *Age and Ageing*, *50*(2), 546–553. 10.1093/ageing/afaa281 33507242 PMC7936020

[CIT0014] Hinchliff, S., Mawson, R. L., Malta, S., & Cliff, G. (2023). How to support the sexual wellbeing of older patients. *BMJ*, *380*, e072388. 10.1136/bmj-2022-072388 36972919

[CIT0015] Hsieh, H.-F., & Shannon, S. E. (2005). Three approaches to qualitative content analysis. *Qualitative Health Research*, *15*(9), 1277–1288. 10.1177/1049732305276687 16204405

[CIT0016] James, H., Nazroo, J., Chatzi, G., & Simpson, P. (2023). How do women and men negotiate sex in later life relationships? A qualitative analysis of data from the English Longitudinal Study of Aging. *Journal of Sex Research*, *60*(9), 1332–1344. 10.1080/00224499.2022.2112934 36043890

[CIT0017] Khan, J., Greaves, E., Tanton, C., Kuper, H., Shakespeare, T., Kpokiri, E., Wang, Y., Ong, J. J., Day, S., Pan, S. W., Tang, W., Wang, B., Peng, X., Liang, B., Zou, H., Tucker, J. D., & Wu, D. (2023). Sexual behaviours and sexual health among middle-aged and older adults in Britain. *Sexually Transmitted Infections*, *99*(3), 173–179. 10.1136/sextrans-2021-055346 35953300 PMC10176408

[CIT0018] King, J., McManus, H., Kwon, A., Gray, R., & McGregor, S. (2022). *HIV, viral hepatitis and sexually transmissible infections in Australia: Annual surveillance report 2022*. The Kirby Institute. 10.26190/SX44-5366

[CIT0019] Kirby Institute (2024). *HIV, viral hepatitis and sexually transmissible infections in Australia: Annual surveillance report 2024: Sexually transmissible infections*. Author.

[CIT0020] Liu, H., Waite, L. J., Shen, S., & Wang, D. H. (2016). Is sex good for your health? A national study on partnered sexuality and cardiovascular risk among older men and women. *Journal of Health and Social Behavior*, *57*(3), 276–296. 10.1177/0022146516661597 27601406 PMC5052677

[CIT0021] Lyons, A., Heywood, W., Fileborn, B., Minichiello, V., Barrett, C., Brown, G., Hinchliff, S., Malta, S., & Crameri, P. (2017). Sexually active older Australian’s knowledge of sexually transmitted infections and safer sexual practices. *Australian and New Zealand Journal of Public Health*, *41*(3), 259–261. 10.1111/1753-6405.12655 28245525

[CIT0022] Lyons, A., Mikolajczak, G., Heywood, W., Fileborn, B., Minichiello, V., Hinchliff, S., Malta, S., Dow, B., Barrett, C., & Brown, G. (2018). Sources of information-seeking on sexually transmitted infections and safer sex by older heterosexual Australian men and women. *Educational Gerontology*, *44*(2–3), 186–195. 10.1080/03601277.2018.1433989

[CIT0023] Malatzky, C., & Hulme, A. (2022). “I love my job…it’s more the systems that we work in”: The challenges encountered by rural sexual and reproductive health practitioners and implications for access to care. *Culture, Health & Sexuality*, *24*(6), 735–749. 10.1080/13691058.2021.1880640 33541254

[CIT0024] Malta, S., Temple‐Smith, M., Bickerstaffe, A., Bourchier, L., & Hocking, J. (2020). “That might be a bit sexy for somebody your age”: Older adult sexual health conversations in primary care. *Australasian Journal on Ageing*, *39 Suppl 1*(S1), 40–48. 10.1111/ajag.12762 32567180

[CIT0025] Malta, S., Temple-Smith, M., Hunter, J., McGavin, D., Lyne, J., Bickerstaffe, A., & Hocking, J. (2018). Could an online or digital aid facilitate discussions about sexual health with older Australians in general practice? *Australian Journal of General Practice*, *47*(12), 870–875. 10.31128/AJGP-04-18-4557 31212407

[CIT0026] Mealing, N. M., Banks, E., Jorm, L. R., Steel, D. G., Clements, M. S., & Rogers, K. D. (2010). Investigation of relative risk estimates from studies of the same population with contrasting response rates and designs. *BMC Medical Research Methodology*, *10*(1), 26. 10.1186/1471-2288-10-26 20356408 PMC2868856

[CIT0027] Menassa, M., Stronks, K., Khatmi, F., Roa Díaz, Z. M., Espinola, O. P., Gamba, M., Itodo, O. A., Buttia, C., Wehrli, F., Minder, B., Velarde, M. R., & Franco, O. H. (2023). Concepts and definitions of healthy ageing: A systematic review and synthesis of theoretical models. *eClinicalMedicine*, *56*(February), 101821. 10.1016/j.eclinm.2022.101821 36684393 PMC9852292

[CIT0028] Nic Giolla Easpaig, B., Reynish, T. D., Hoang, H., Bridgman, H., Corvinus-Jones, S. L., & Auckland, S. (2022). A systematic review of the health and health care of rural sexual and gender minorities in the UK, USA, Canada, Australia and New Zealand. *Rural and Remote Health*, *22*(3), 6999. 10.22605/RRH6999 35794784

[CIT0029] Roman Lay, A. A., De Oliveira Duarte, Y. A., Duarte, L. S., & Vilela Borges, A. L. (2023). Sexual activity and satisfaction in older adults from a Brazilian cohort study. *Aging & Mental Health*, *27*(2), 417–424. 10.1080/13607863.2021.2025338 35023418

[CIT0030] Shen, S., & Liu, H. (2023). Is sex good for your brain? A national longitudinal study on sexuality and cognitive function among older adults in the United States. *Journal of Sex Research*, *60*(9), 1345–1355. 10.1080/00224499.2023.2238257 37506374 PMC10615694

[CIT0031] Sinković, M., & Towler, L. (2019). Sexual aging: A systematic review of qualitative research on the sexuality and sexual health of older adults. *Qualitative Health Research*, *29*(9), 1239–1254. 10.1177/1049732318819834 30584788

[CIT0032] Stahl, M., Kate, A., Gale, J., Lewis, D. C., & Kleiber, D. (2019). Pathways to pleasure: Older adult women’s reflections on being sexual beings. *Journal of Women & Aging*, *31*(1), 30–48. 10.1080/08952841.2017.1409305 29210621

[CIT0033] Stentagg, M., Skär, L., Berglund, J. S., & Lindberg, T. (2021). Cross-sectional study of sexual activity and satisfaction among older adult’s ≥60 years of age. *Sexual Medicine*, *9*(2), 100316. 10.1016/j.esxm.2020.100316 33676227 PMC8072140

[CIT0034] Thomas, J., McCosker, A., Parkinson, S., Hegarty, K., Featherstone, D., Kennedy, J., Holcombe-James, I., Ormond-Parker, L., & Ganley, L. (2023). *Measuring Australia’s digital divide: Australian digital inclusion index: 2023*. ARC Centre of Excellence for Automated Decision-Making and Society, RMIT University, Swinburne University of Technology, and Telstra. https://www.digitalinclusionindex.org.au/

[CIT0035] Towler, L. B., Graham, C. A., Bishop, F. L., & Hinchliff, S. (2022). Sex and relationships in later life: Older adults’ experiences and perceptions of sexual changes. *The Journal of Sex Research*, *60*(9), 1318–1331. 10.1080/00224499.2022.2093322 35852483

[CIT0036] United Nations, processed by Our World in Data (2024). Sex ratio by age—UN WPP, world population prospects. https://ourworldindata.org/grapher/sex-ratio-by-age

[CIT0037] WHO (2006). *Defining sexual health report of a technical consultation on sexual health 28–31 January 2002*. Author.

[CIT0038] WHO (2020). *Decade of healthy ageing: Plan of action*. Author. https://www.who.int/publications/m/item/decade-of-healthy-ageing-plan-of-action

[CIT0039] Zhang, Y., & Liu, H. (2020). A national longitudinal study of partnered sex, relationship quality, and mental health among older adults. *The Journals of Gerontology: Series B*, *75*(8), 1772–1782. 10.1093/geronb/gbz074 PMC7489086 31132123

